# A Comparison of Weight-Related Behaviors of Hispanic Mothers and Children by Acculturation Level

**DOI:** 10.3390/ijerph18020503

**Published:** 2021-01-09

**Authors:** Colleen L. Delaney, Kim Spaccarotella, Virginia Quick, Carol Byrd-Bredbenner

**Affiliations:** 1 Department of Nutritional Science, Rutgers University, New Brunswick, NJ 08901-8554, USA; vquick@njaes.rutgers.edu (V.Q.); bredbenner@sebs.rutgers.edu (C.B.-B.); 2 Department of Biological Sciences, Kean University, Union, NJ 07083-7131, USA; kspaccar@kean.edu

**Keywords:** acculturation, Hispanic, mothers, nutrition, home environment, behavior

## Abstract

Hispanic mothers and children in the United States experience a high prevalence of obesity, which may be affected by maternal acculturation level. Little is known about the association of acculturation on weight-related behaviors. This study describes differences in weight-related behaviors by acculturation level of Hispanic mothers residing in the U.S. and compares them to behaviors of White mothers. Acculturation level was determined using personal acculturation and acculturation environment variables. Cluster analysis of acculturation variables identified three groups of Hispanic mothers: low personal and environmental acculturation (*n* = 46), high personal and low environmental acculturation (*n* = 65), and high personal and environmental acculturation (*n* = 38). Results indicate that, compared to White mothers (*n* = 340), the least acculturated cluster of Hispanic mothers tended to model physical activity less often and the most acculturated exerted more pressure on children to eat. Mothers in the least acculturated cluster tended to rate children’s health status lower, indicate that children had greater fruit juice and less milk intakes, have more meals in locations associated with less healthy meals, and have the least space and supports for physical activity. Findings highlight relationships between maternal acculturation level and weight-related behaviors and suggest strategies for helping acculturating Hispanic mothers create healthier lifestyles and home environments.

## 1. Introduction

Individuals of Hispanic origin make up the largest ethnic group in the U.S. [1]—accounting for 17% of the population. These individuals experience disproportionate health disparities compared to most other racial and ethnic groups [2]. Of the top leading causes of deaths for Hispanic persons in the U.S., most are diet related (e.g., cancer, heart disease, diabetes, liver and kidney disease) [3]. Many of these health conditions have strong links with obesity [4,5,6,7,8].

Obesity prevalence in the U.S. is rising, affecting 42% of the population; racial and ethnic disparities are seen in obesity, with prevalence being highest in Hispanic and non-Hispanic Black adults (i.e., 45% and 50%, respectively) [9]. Hispanic children in the U.S. have the highest rates of overweight among all racial and ethnic groups, with 26% being obese versus 22%, 14%, and 11% of non-Hispanic Black, White, and Asian children, respectively [10]. This weight disparity emerges at a young age—22% of preschool-aged Hispanic children in the U.S. are obese compared to 8% of all other U.S. preschool-aged children and 5% of preschoolers living in Mexico classified as moderately to severely overweight [11,12].

A factor complicating the study of Hispanic individuals is the range of acculturation levels or extent of cultural and psychological transition in this population. Approximately one-third of all Hispanic individuals living in the U.S. are foreign born, originating predominately from Mexico, Puerto Rico, and Cuba [13,14,15]. Foreign-born individuals undergo the process of acculturation as they interact with the culture of the country of migration [16,17]. The acculturation transition can affect all aspects of their lives, including diet and health behaviors [16,17,18]. For example, lower levels of acculturation among Hispanic populations are associated with increased obesity and overweight risk, perhaps because of immigration-associated stress and changes in lifestyle practices and home environments [19,20,21,22,23]. Dietary acculturation refers to changes in diet-related behaviors that occur as acculturation level increases. Dietary patterns generally tend to transition from traditional food habits associated with the country of origin to those of the new country of residence [24]. For instance, Mexican immigrants to the U.S. who are in the earliest stages of acculturation tend to eat a more traditional diet, which is rich in fruits, vegetables, and fiber, and low in saturated fat and simple sugars [25]. In contrast, the dietary patterns of those who are more acculturated tend to be more reflective of the prevailing culture, such as more highly processed convenience foods, more fast foods, and fewer fruits and vegetables [25]. Increasing dietary acculturation has been linked with greater risk of poor health outcomes, including obesity and co-morbidities, such as diabetes [13,25,26]. Other changes associated with increasing acculturation that affect health outcomes include decreased physical activity, shorter sleep duration, and changes in eating patterns [27,28,29].

Dietary acculturation can be influenced by psychosocial and environmental factors [30]. Traditional psychosocial characteristics pertaining to mealtime may play a protective role in Hispanic individuals who are less acculturated, such as the belief in the central role of the family, *familismo*, that undergirds socialization. This strong belief in the importance of the family unit influences behaviors through modeling and expression of behavioral expectations, such as gathering at mealtime and eating traditional foods, which in turn helps supports intake of fresh foods vs. processed foods, more frequent home prepared meals, and fewer meals eaten outside of the home [30]. Environmental factors that play a role in dietary acculturation include access and availability of traditional foods, social support for maintaining traditional eating patterns or accepting new patterns, and time available for food preparation and exercise [30,31,32].

Acculturation research tends to focus on personal acculturation of individuals and considers factors such as language acquisition and preference, ethnic identification, and length of residency in the host culture [16,33,34,35,36]. Despite its importance, little attention has been given to the ‘acculturation environment’—environmental factors external to the individual that influence the acculturation process [37]. Environmental factors that affect acculturation include aspects of the neighborhood where individuals reside, such as population density of recent immigrants, the predominant language of their neighborhood, and access to stores that stock mainstream vs. traditional goods, as well as language used in prevailing media [37].

In recent years, significant research has focused on identifying environmental, social, and personal factors associated with increased obesity risk and developing interventions to address these factors [38,39,40,41]. Interventions targeted to specific audiences are associated with greater acceptance and application of intervention messages [42]. Even though the Hispanic population residing in the U.S. is sizable and is disproportionately affected by obesity and overweight and related comorbidities, few obesity prevention interventions have been targeted and tailored to this audience [43]. Additionally, to the authors’ knowledge, none have considered differences in acculturation among this audience.

A better understanding of the associations among acculturation level and weight-related behaviors could lead to the development of interventions with greater potential for reducing obesity and overweight risk among the Hispanic adults and children in the U.S. [21,28,44,45,46,47,48,49,50,51,52,53]. Therefore, the purpose of this study was to describe the differences in weight-related behaviors by acculturation level of Hispanic mothers of young children residing in the U.S. and compare them to White mothers with young children who served as an acculturated standard.

## 2. Materials and Methods

The study protocol (11-294Mc) was approved by the Rutgers University Institutional Review Board. This study uses the baseline data from the HomeStyles randomized control trial, which used the Social Cognitive Theory and a social-ecological framework to investigate environmental, social, and personal characteristics of home environments associated with health and body weight [38,41]. Outcomes have been reported elsewhere for a subset of study participants [38,39,41,54]. Participant recruitment occurred using a variety of methods, in both English and Spanish, including word of mouth, outreach at community events, and electronic postings that invited parents to join a program to help them “build even happier, healthier, safer families”.

### 2.1. Participants

To be eligible to complete the baseline survey, individuals had to have at least one child aged 2 to <9 years, be between the ages of 20 and 45 years, make all or most household food purchasing and preparation decisions, live in the study target areas (New Jersey or Arizona), give informed consent, and complete the baseline survey. Participants received a $15 stipend. Participants who replied to recruitment announcements (*n* = 5495) were not included in the data set analyzed in this study if they did not complete the study screener (*n* = 217), did not consent (*n* = 405), did not meet eligibility criteria (*n* = 3343), failed to complete the baseline survey (*n* = 862), or gave implausible responses (e.g., answered many questions similarly; *n* = 34). In addition, those not pertinent to the purpose of this secondary analysis (i.e., males [*n* = 49] and those who were of a race/ethnicity that was neither White nor Hispanic [*n* = 95]) were culled from the data set. The final analytic sample was 489.

### 2.2. Survey Instrument

The “**H**ome **O**besogenicity **M**easure of **E**nvironment**S**” (HOMES) baseline survey was used to collect data. To summarize the previously reported details of this survey [38,40], it was based on a comprehensive literature review that identified behavior and environmental variables associated with weight status in parents and young children along with validated, reliable, relevant scales for assessing these variables [38,40]. When multiple scales assessing a variable were located, a panel of experts in nutrition and survey methods reviewed them to identify the scale that had the greatest reliability, validity, and relevance to the study and was easy for participants to complete and researchers to administer and score. In cases where a suitable scale for assessing a variable was not available, a new scale was developed using standard practices for scale development; that is, items were developed and reviewed by experts for content validity, tested cognitively with parents of young children, pilot tested, pretested, and field tested. At each stage of development, the findings were subjected to expert review and refined [40,55,56,57]. All participants in the survey development stages had the same characteristics as those who completed the HOMES baseline study but did not participate in the baseline study.

The HOMES baseline survey gathered data on demographic characteristics (e.g., age, education, race/ethnicity, total parents in the household, maternal employment, family affluence), food insecurity risk, maternal health status, maternal weight-related behaviors, and parenting practices. Mothers also reported the health status and weight-related behaviors for one of their children. Mothers with 2 or more children between the ages of 2 and <9 years were directed to provide data on one child born that was closest to a randomly chosen time and date (i.e., noon on 1 June). Assessments of the home environment were family mealtime importance, frequency, and location; household food availability; physical activity space and supports; and neighborhood safety.

Race/ethnicity was reported as White, Hispanic, Black, Asian, Native American, or American Indian, and/or Alaskan Native or Pacific Islander; this item was used to identify the participants for this secondary analysis. The Family Affluence Scale, a reliable marker of socioeconomic status [58,59], has 4 items that generate a family affluence score of 0 to 9, with higher scores indicating more affluence [58,59]. Food insecurity risk was evaluated with the 2-item scale developed by Hager et al. [60]. Maternal and child health status were appraised using the 5-point (poor to excellent) health rating item from the Health-Related Quality of Life Scale developed by the Centers for Disease Control and Prevention [61].

Mother and child physical activity levels were evaluated using the 3-item HOMES Physical Activity Questionnaire level [38,62,63], which measured days per week spent walking, doing moderate activity, and doing vigorous activity. An indicator of sedentary behavior of mothers and children was determined using total minutes spent watching television, movies, and videos, and using computers each day. The Block Fruit/Vegetable Screener determined mothers’ consumption of fruits and vegetables daily and children’s intake of fruit/vegetable juice each day [64,65,66]. The HOMES Drinks Intake Screener estimated daily intake of sugar-sweetened beverages, such as soft drinks, fruit drinks, tea, and coffee by mothers and children [38,67,68]. The drinks screener also determined children’s daily milk intake [38,67,68].

Weight-related parenting practices included maternal modeling of behaviors and their child feeding practices. The Modeling of Physical Activity scale assessed how frequently mothers actively played with their children [38,40,62]. The Modeling of Healthy Eating scale evaluated how important mothers felt it was to engage in this behavior, with answers being a 5-point Likert agreement scale ranging from strongly disagree to strongly agree [38,40,62]. Child feeding practices evaluated included using food to reward children for eating healthy foods, pressuring children to eat, and controlling food intake amounts of children [62,69,70,71,72,73,74,75]. These child feeding practices scales used a 5-point Likert agreement scale ranging from strongly disagree to strongly agree. Higher scores on all weight-related parenting practices scales indicate greater use of the practice.

Characteristics of the home environment assessed included the value mothers placed on family meals using a 5-point Likert agreement scale ranging from strongly disagree to strongly agree. Mothers also reported the total number of family meals eaten each week as well as how many days per week family meals were consumed in locations associated with healthier meals (i.e., dining table) and less healthy meals (i.e., car, fast food restaurant, in from of a television) [76,77,78]. The amount of fruits/vegetables and sugar-sweetened beverages available in the home was determined with a household food supply frequency questionnaire [38]. The Hop-Up questionnaire appraised the space and supports available for physical activity inside the home, in the outdoor/yard area right outside the home, and in the neighborhood as well as overall neighborhood safety [79,80].

As acculturation is an abstract construct that is fluid in nature, it can be difficult to define and measure [47]. Proxy variables, such as language use or nativity, can be used to estimate acculturation [81]. The strongest single indicator of acculturation tends to be language use or preference [82]. In this study, personal and environmental acculturation were assessed for those reporting Hispanic as their race/ethnicity. Personal acculturation was assessed using these 3 variables: language chosen to complete the survey, language most commonly spoken at home, and participant’s country of birth [34,35,36,37,83,84,85]. Each personal variable was dichotomously coded with those who completed the survey in English, spoke English at home, and/or were born in the U.S. being considered more acculturated while the opposite was considered less acculturated. Acculturation environment variable information were derived from statistics geocoded as census tract data using the U.S. postal zip code of each participant’s home residence [37]. Acculturation environment variables in each participant’s home census tract were: percentage of foreign-born individuals, percentage of foreign-born individuals arriving within 5 years prior to the census, and percentage of Spanish-speaking households reporting speaking English less than very well [37]. Acculturation environment variables were standardized by state (New Jersey or Arizona) by dichotomously coding each variable as more acculturated if the percentage was at or above the median threshold for the participant’s state of residence and less acculturated if the percentage value was below the median threshold. For example, a person who lived in a neighborhood with a higher percentage of foreign-born individuals than the state’s median was coded as less acculturated for this variable.

### 2.3. Data Analysis

Each of the dichotomously coded personal acculturation and acculturation environment measures were included in Ward’s Hierarchical Cluster Analysis to partition Hispanic participants into meaningful acculturation groups. Cluster analysis allows the relationships among the personal and environment measures to be explored while accounting for the complex, latent interactions among these measures. The goal of cluster analysis is to merge individuals into similar groups that maximize within-group homogeneity and between-group heterogeneity [86]. Ward’s hierarchical method was selected because it determines the total number of clusters in circumstances where the number of clusters is unknown, as was the case in this study [86]. Descriptive statistics (e.g., means, standard deviations, confidence intervals) were computed to describe the participants and scale scores. Differences in weight-related behaviors were compared among and between study groups (i.e., Hispanic acculturation cluster groups and non-Hispanic White participants) using analysis of covariance (ANCOVA) and Bonferroni post-hoc tests, with family affluence score serving as the covariate because family socioeconomic status is thought to be associated with differences related to acculturation [25,87,88,89,90,91,92,93,94,95]. Family affluence was compared among and between study groups using Analysis of Variance (ANOVA) and Tukey post-hoc tests. Effect size was calculated as partial eta-squared values, with 0.01, 0.06, and 0.14 serving as the thresholds for small, medium, and large effect sizes, respectively [96]. Due to the multiple comparisons, the probability level for the main effects was set at *p* ≤ 0.01 to reduce the risk of type I errors. Post-hoc probability was set to *p* < 0.05. Analyses were conducted with SPSS software version 27.0 (IBM Corporation, Chicago, IL, USA).

## 3. Results

The final analytic sample of 489 mothers was comprised of 340 who were non-Hispanic White and 149 who were Hispanic. The scree plot and agglomeration schedule generated from Ward’s hierarchical cluster methodology indicated that Hispanic mothers grouped into three acculturation groups. An examination of the personal acculturation and acculturation environment scores indicated that Hispanic mothers assigned to Cluster 1 (*n* = 46) were the least acculturated, achieving a total acculturation score of 1.7 on a 6-point scale (Table 1). As shown in Table 2, most completed the survey in Spanish, spoke Spanish at home, and were born outside the United States. Cluster 1 mothers also lived in areas where the percent of foreign-born individuals, percent of foreign-born individuals arriving between 2010 and 2015, and percent of households speaking English less than very well were at or above the median. Cluster 2 (*n* = 65) was somewhat acculturated, having a total acculturation score of 3.5 out of 6. Cluster 2 Hispanic mothers were similar to Cluster 1 regarding environmental acculturation measures but differed on personal acculturation measures in that all or nearly all completed the survey in English, spoke English at homes, and were born in the United States. That is, Cluster 2 lived in low acculturation environments but had higher personal acculturation. Cluster 3 (*n* = 48) Hispanic mothers were the most acculturated, achieving a mean acculturation score of 5.37 on the 6-point scale. Most Cluster 3 mothers lived in areas where percent of foreign-born individuals, percent of foreign-born individuals arriving between 2010 and 2015, and percent of households speaking English less than very well were below the median. Like the somewhat acculturated mothers in Cluster 2, all mothers in Cluster 3 completed the survey in English, spoke English in their homes and were born in the U.S. Thus, Cluster 3 lived in high acculturation environments and were personally acculturated. ANOVA and follow-up procedures comparing total acculturation scores by cluster revealed significant differences for all pairwise comparisons of clusters, indicating that each cluster uniquely represented a different level of acculturation.

Mothers were of moderate family affluence. ANOVA and post-hoc test results indicated that White mothers and Cluster 3 mothers did not differ; however, White mothers had significantly greater family affluence than both Clusters 1 and 2, while Cluster 3 mothers had significantly greater family affluence than Cluster 1 mothers. These differences in family affluence confirmed the need for family affluence to serve as a covariate in subsequent analyses.

As shown in Table 3, results from ANCOVA and post-hoc pairwise comparisons of mothers indicated that they tended to be similar regarding age, education level, number of parents in the household, and food insecurity risk. Employment status differed significantly among study groups, with mothers in the least acculturated cluster (Cluster 1) being less likely to have paid employment than all other groups of mothers. However, effect sizes for all demographic characteristic differences were small.

Mothers reported good to very good health status, with no groups differing significantly (Table 4). Mothers’ weight-related behaviors were similar across groups. Few significant among- and between-group differences were noted in mothers’ weight-related parenting practices and child feeding practices. White mothers tended to model physical activity more often than Hispanic mothers in Cluster 1, and the most acculturated Hispanic mothers (Cluster 3) tended to pressure children to eat more than White mothers.

Children’s health status was very good to excellent, with Hispanic mothers in the two least acculturated groups (Clusters 1 and 2) rating child health significantly lower than White mothers. ANCOVA of children’s weight-related behaviors indicated that physical activity and screentime behaviors did not significantly differ among groups. However, beverage intake of children was significantly different among study groups. That is, children of Hispanic mothers in Cluster 2 drank significantly more sugar-sweetened beverages than the least acculturated Hispanic mothers (Cluster 1) and White mothers. Children of Hispanic mothers in Cluster 1 tended to drink significantly less milk and more fruit juice than children in other groups. However, the effect sizes for all child assessments were small.

The home environment characteristics described in Table 5 indicate no significant differences among study groups in the importance placed on family meals or frequency of family meals. However, family meal locations differed significantly among study groups, with the least acculturated Hispanic mothers in Cluster 1 tending to more meals eaten in locations associated with less healthy meals, such as cars and in front of the TV than other groups and fewer meals at dining tables. Availability of fruits/vegetables and sugar-sweetened beverages in the household did not differ significantly among and between groups. Hispanic mothers in Cluster 1 tended to have less home and outdoor/yard space and supports for physical activity, with these differences being significantly lower in comparison to White mothers. The two less acculturated clusters of Hispanic mothers (Clusters 1 and 2) reported significantly lower neighborhood safety than the most acculturated Hispanic mothers as well as White mothers. Effect sizes for home environment characteristics were small, except for frequency of eating family meals in cars, which had a large effect size.

## 4. Discussion

The findings of this study indicate that using personal acculturation and acculturation environment variables in a cluster analysis generated three distinctly different acculturation clusters of Hispanic mothers. Cluster 1 had the lowest personal acculturation. Both Clusters 1 and 2 lived in less acculturated neighborhoods in contrast to Cluster 3 mothers who lived in acculturated neighborhoods. Clusters 2 and 3 had high personal acculturation. It is interesting to note there was not a cluster with low personal acculturation and acculturation environment was not found—this is likely a reflection of the impact a high acculturation environment has on the progression of personal acculturation characteristics [97]. The observation that White and the most acculturated cluster of Hispanic mothers had similar family affluence, coupled with the significant differences between these two groups and the two less acculturated clusters for family affluence, supports previous research reporting that socioeconomic status increases with acculturation [25,87,88,89,90,91,92,93,94,95]. The lower rate of employment of the least acculturated Hispanic mothers may be due to lack of facility with the English language, lack of cultural capital (e.g., knowledge of societal customs and valued attitudes and behaviors), lack of social capital (i.e., inclusion in social networks that provide instrumental relationships and offer access to resources, such as employment opportunities), and neighborhood environments with limited employment opportunities [87,98,99,100,101,102,103,104,105,106,107]. However, given that the least acculturated Hispanic mothers (Cluster 1) differed significantly from all other groups, including Cluster 2 which had a similar acculturation environment, the findings appear to indicate that personal acculturation factors, such as ability to use the prevailing language, play a central role in employment status.

Study findings suggest that mothers are more alike than different regardless of their acculturation level and health status. However, Hispanic mothers in the two less acculturated clusters reported a significantly lower health status for their children than White mothers. These data suggest that acculturation level is positively linked to child health status. This supports previous research indicating Hispanic mothers with lower acculturation tend to have children with poorer health [108,109], which could be related to lack of health insurance or access to health care facilities in less acculturated neighborhoods [110,111].

Mothers nearly met the recommended intake of fruits and vegetables (five or more servings per day) [112] and reported low intake of sugar-sweetened beverages. Although mothers had similar dietary intake, they reported significant differences in their children’s intake. Children of the least acculturated Hispanic mothers, like those of White mothers, had the lowest intakes of sugar-sweetened beverages, yet these children of Cluster 1 Hispanic mothers consumed more 100% fruit juice and less milk than White and more acculturated clusters. Although it is not clear why these differences occurred, it is possible that the least acculturated mothers were not able unable to discern the difference between 100% fruit juice and fruit-flavored sugar-sweetened beverages because sugar-sweetened beverages often feature pictures of fruit on the label, yet contain no real fruit juice. These images can lead to confusion regarding the fruit content of the product, especially among those with less English language facility [113,114]. The least acculturated Hispanic mothers had a lower family affluence score, and thus, were more likely to participate in the Special Supplemental Feeding Program for Women, Infants, and Children (WIC) which would increase their access to juice [115]. Indeed, WIC participants tend to report higher intakes of juice and sugar-sweetened beverages than non-WIC participants, but an intake of milk similar to non-WIC families was not observed in this study [116]. The lower milk intake by children of the least acculturated Hispanic mothers could be due to the relatively lower intake of fluid milk in many Hispanic groups, such as those from Mexico and Central America, due to the prevalence of lactose intolerance and the higher prices of milk seen in less acculturated neighborhoods in the U.S. [117,118].

Study findings suggest that acculturation was not associated with mothers’ own diets, but mothers’ acculturation played a role in children’s intake. These findings contrast with previous research indicating that acculturation is linked with adult diets [24,30,31], but the acculturation level of children’s caregivers contributes little to the dietary intake of young children in their care [53]. Although the reason for these differences is not known, it is important to consider that previous studies tend to consider only personal acculturation characteristics, unlike this study which included both personal and environment acculturation characteristics.

Mothers and children in all groups had low physical activity scores and exceeded the recommendations for screentime (<1 h daily of high-quality programming for children older than 2 years) [119]. Although differences among groups were not significant, the least acculturated mothers and children tended to have the lowest physical activity and modeled physical through co-play with children the least. This likely is because they also had less access to space and supports for physical activity within their homes, yards, and neighborhoods and lower neighborhood safety, suggesting that acculturation environment limited access to amenities that promote physical activity [120,121,122,123,124,125,126]. Similarly, Cluster 2 who also lived in a low acculturation environment reported lower neighborhood safety.

The frequency of family meals eaten at less healthy locations tended to decrease and meals at healthy locations rise as acculturation level increased. The frequent consumption of family meals in cars by the least acculturated cluster may be a time management response to the shift work schedules and unskilled labor job types common to this population [31,44,106,107]. In addition, the frequent meals in cars may reflect limited space and equipment within homes for preparing, storing, and/or eating foods thereby making it convenient and necessary to frequently eat meals outside of the home [127]. Living space also may be constrained to the point that it is not possible to accommodate a dining table [127,128], so meals are eaten in front of the television. Despite the frequent, on-the-go eating of less acculturated families, mothers reported adequate (at least five servings/person/day) availability of fruits/vegetables in their homes [112].

This study supports previous findings that acculturation is associated with behaviors [24,25,27,89,90,92,93,94] and home environment characteristics [120,121,122,123,124,125,127] that impact weight status and demonstrates that both personal and environmental factors contribute to links among acculturation [37], independent of family affluence, and lifestyles and home environments of mothers with young children. The results highlight key similarities and differences in health status and weight-related behaviors of Hispanic mothers and their young children based on maternal acculturation level. Findings suggest that interventions for reducing obesity and overweight risk among both Hispanic and White adults and children in the U.S., regardless of Hispanic mothers’ acculturation level, should focus on increasing physical activity, reducing screentime, and increasing the frequency of maternal modeling of healthy behaviors. For audiences who have lower personal acculturation, strategies for differentiating between sugar-sweetened fruit drinks and 100% fruit juice may be needed. Strategies for successfully addressing the barriers in low acculturation environments to healthy behaviors, such as eating at a dining table rather than locations associated with less healthy meals, easing access to health care facilities, increasing availability of physical activity space and supports, improving safety perceptions of neighborhoods, and possibly changing labeling of sugar-sweetened fruit drinks to make it easier to determine these are not fruit juice equivalents, also are needed.

This study is among the first to examine the weight-related behaviors of Hispanic mothers of young children by acculturation level. It is important to note that the findings of this study differ from the narrative that acculturation unequivocally leads to negative health behaviors and health consequences among Hispanic populations, even finding some benefit to acculturation in this population. In addition, it is the first known to include acculturation environment variables in the determination of mothers’ acculturation level. The strengths of this study include the use of valid, reliable scales in the survey and overall large sample size, although the number of participants in each cluster was more modest. This study is limited by the potential for social desirability bias and self-reporting of data. The cross-sectional nature of this study also negates the possibility of inferring causal relationships. Additionally, the race/ethnicity of spouses/partners was not available and should be considered in future studies.

## 5. Conclusions

Overall, findings suggest weight-related behaviors that educational interventions for this population should target and highlight the important roles of considering maternal acculturation when developing interventions aiming to help acculturating Hispanic mothers of young children create healthier lifestyles and home environments. For instance, interventions could focus on encouraging families to share meals at home together or develop strategies for accessing safe locations to engage in physical activity with their children. Clarifying how acculturation is linked to health behaviors can lead to health promotion materials that are tailored to and perhaps resonate better with acculturating Hispanic audiences, thereby contributing to the reduction in health disparities that are so prevalent in this population. Future research should investigate whether the findings of this study hold true for other immigrant populations undergoing acculturation in the United States and other nations.

## Figures and Tables

**Table 1:** Acculturation and Family Affluence of Mothers (*n* = 489).

Characteristic	Hispanic Mothers’ Cluster ^1^			White Mothers	*F df* = *3, 485* *	ANOVA # *p*	Partial Eta-Squared
	Cluster 1	Cluster 2	Cluster 3				
	(*n* = 46)	(*n* = 65)	(*n* = 38)	(*n* = 340)			
	Mean ± SD	Mean ± SD	Mean ± SD	Mean ± SD			
	(95% CI ^†^)	(95% CI ^†^)	(95% CI ^†^)	(95% CI ^†^)			
Acculturation Level ^2^	1.70 ± 1.31	3.50 ± 0.56	5.37 ± 0.63	--	182.051	<0.0001 ^ABC^	0.793
	(1.31, 2.09)	(3.35, 3.63)	(5.16, 5.58)				
Personal Acculturation ^3^	0.72 ± 0.86	2.88 ± 0.33	3.00 ± 0.00	--	280.409	<0.0001 ^AC^	0.497
	(0.46, 0.97)	(2.79, 2.96)	(3.00, 3.00)				
Acculturation Environment ^4^	0.98 ± 0.95	0.62 ± 0.57	2.37 ± 0.63	--	72.105	<0.0001 ^ABC^	0.714
	(0.69, 1.26)	(0.47, 0.76)	(2.16, 2.58)				
Family Affluence Score ^5^	4.57 ± 1.66	4.98 ± 1.88	5.76 ± 1.70	5.59 ± 1.73	6.676	<0.0001 ^BDE^	0.040
	(4.07, 5.06)	(4.52, 5.45)	(5.20, 6.32)	(5.41, 5.78)			

* df = Degrees of Freedom; # Analysis of Variance (ANOVA). Superscript capital letters indicate significant (*p* < 0.05) Tukey post-hoc tests results: ^A^ Cluster 1 and Cluster 2, ^B^ Cluster 1 and Cluster 3, ^C^ Cluster 2 and Cluster 3, ^D^ Cluster 1 and White, ^E^ Cluster 2 and White, and ^F^ Cluster 3 and White. ^†^ CI = confidence interval. ^1^ Ward’s Hierarchical Cluster Analysis was used to assign Hispanic mothers to clusters based on acculturation (i.e., three personal measures [language of survey, language used in home, country of birth] and three environmental measures based on census tract [% foreign-born individuals, % foreign-born individuals arriving within the period 2010–2015, % Spanish-speaking households speaking English less than very well]). ^2^ Acculturation level scale contains six items; scores range from 0 to 6; higher scores indicate higher acculturation level. ^3^ Personal Acculturation scale contains three items; scores range from 0 to 3; higher scores indicate higher personal acculturation. ^4^ Acculturation environment contains three items; scores range from 0 to 3; higher scores indicate higher acculturation environments. ^5^ Family Affluence Scale contains four items; scores range from 0 to 9; higher scores indicate greater family affluence.

**Table 2:** Hispanic Mothers Clustered by Acculturation Variables (*n* = 149).

Acculturation Variable	Hispanic Mothers’ Cluster ^1^			F df = 2, 146 *	ANOVA # *p*	Partial Eta-Squared
	Cluster 1 (*n* = 46) *n* (%)	Cluster 2 (*n* = 65) *n* (%)	Cluster 3 (*n* = 38) *n* (%)			
**Personal Acculturation Variables**						
**Language of Survey**				71.711	<0.001 ^AB^	0.496
English	19 (41.30%)	65 (100.00%)	38 (100.00%)			
Spanish	27 (58.70%)	0 (0.00%)	0 (0.00%)			
**Language used in Home**				410.445	<0.001 ^AB^	0.849
English	3 (6.52%)	63 (96.92%)	38 (100.00%)			
Spanish	43 (93.48%)	2 (3.08%)	0 (0.00%)			
**Country of Birth**				84.026	<0.001 ^AB^	0.535
U.S.	11 (23.91%)	59 (90.77%)	38 (100.00%)			
Outside of U.S.	35 (76.09%)	6 (9.23%)	0 (0.00%)			
**Acculturation Environment Variables**						
**% Foreign-Born Individuals in Postal Zip Code**				189.563	<0.001 ^ABC^	0.722
Below Median	13 (28.26%)	0 (0.00%)	38 (100.00%)			
At or Above Median	33 (71.74%)	65 (100.00%)	0 (0.00%)			
**% Foreign-Born Individuals Arriving Within 2010–2015 in Postal Zip Code**				2.460	0.089	0.033
Below Median	22 (47.83%)	31 (47.69%)	26 (68.42%)			
At or Above Median	24 (52.17%)	34 (52.31%)	12 (31.58%)			
**% Spanish-Speaking Households Speaking English Less than Very Well in Postal Zip Code**				23.378	<0.001 ^BC^	0.243
Below Median	10 (21.74%)	9 (13.85%)	26 (68.42%)			
At or Above Median	36 (78.26%)	56 (86.15%)	12 (31.58%)			

* df = Degrees of Freedom; # Analysis of Variance (ANOVA). Superscript capital letters indicate significant (*p* < 0.05) Tukey post-hoc tests results: ^A^ Cluster 1 and Cluster 2, ^B^ Cluster 1 and Cluster 3, and ^C^ Cluster 2 and Cluster 3. ^1^ Ward’s Hierarchical Cluster Analysis was used to assign Hispanic mothers to clusters based on acculturation (i.e., three personal measures [language of survey, language used in home, country of birth] and three environmental measures based on census tract [%foreign-born individuals, % foreign-born individuals arriving within the period 2010–2015, % Spanish-speaking households speaking English less than very well]).

**Table 3:** Demographic Characteristics of Mothers (*n* = 489).

Characteristic	Hispanic Mothers’ Cluster ^1^			White Mothers	*F df = 3, 485* *	ANCOVA # *p*	Partial Eta-Squared
	Cluster 1 (*n* = 46)	Cluster 2 (*n* = 65)	Cluster 3 (*n* = 38)	(*n* = 340)			
	Mean ± SD	Mean ± SD	Mean ± SD	Mean ± SD			
	(95% CI ^†^)	(95% CI ^†^)	(95% CI ^†^)	(95% CI ^†^)			
Age (years)	31.13 ± 6.07	30.26 ± 5.18	31.05 ± 5.64	33.33 ± 5.44	6.031	<0.0001 ^E^	0.047
	(29.33, 32.93)	(28.98, 31.54)	(29.20, 32.91)	(32.75, 33.91)			
Maternal Education ^2^	1.98 ± 0.71	2.11 ± 0.73	2.45 ± 0.55	2.39 ± 0.71	3.579	0.014	0.042
	(1.77, 2.19)	(1.93, 2.29)	(2.27, 2.63)	(2.31, 2.46)			
Food Insecurity Risk ^3^	1.89 ± 0.88	1.85 ± 0.94	1.97 ± 0.94	1.72 ± 0.95	1.164	0.323	0.008
	(1.63, 2.15)	(1.61, 2.08)	(1.66, 2.28)	(1.62, 1.82)			
Parents in Household ^4^	1.83 ± 0.38	1.68 ± 0.35	1.74 ± 0.45	1.86 ± 0.35	4.512	0.004 ^E^	0.031
	(1.71, 1.94)	(1.56, 1.79)	(1.59, 1.88)	(1.82, 1.90)			
Maternal Employment Status ^5^	1.61 ± 0.83	2.22 ± 0.94	2.21 ± 0.93	2.11 ± 0.90	4.668	0.003 ^ABD^	0.030
	(1.36, 1.86)	(1.98, 2.45)	(1.90, 2.52)	(2.01, 2.20)			

* df = Degrees of Freedom; # Analysis of Covariance (ANCOVA) with family affluence score as the covariate. Superscript capital letters indicate significant (*p* < 0.05) between-group differences using Bonferroni post-hoc tests: ^A^ Cluster 1 and Cluster 2, ^B^ Cluster 1 and Cluster 3, ^C^ Cluster 2 and Cluster 3, ^D^ Cluster 1 and White, ^E^ Cluster 2 and White, and ^F^ Cluster 3 and White. ^†^ CI = confidence interval. ^1^ Ward’s Hierarchical Cluster Analysis was used to assign Hispanic mothers to clusters based on acculturation (i.e., three personal measures [language of survey, language used in home, country of birth] and three environmental measures based on census tract [% foreign-born individuals, % foreign-born individuals arriving within the period 2010–2015, % Spanish-speaking households speaking English less than very well]). ^2^ Education: high school or less, some college or associate degree, bachelor’s degree or higher; scored 1 to 3, respectively. ^3^ Food insecurity risk scale: possible score range = 1 to 4; higher scores indicate greater risk of food insecurity [1]. ^4^ Parents in household: possible score range = 1 to 2. ^5^ Employment: possible score range = 1 to 3; 1 = does not work, 2 = works part time, and 3 = works full time.

**Table 4:** Mother and Child Health and Weight-Related Behaviors (*n* = 489).

Characteristic	Hispanic Mothers’ Cluster ^1^			White Mothers	*F df = 3, 485* *	ANCOVA #	Partial Eta-Squared
	Cluster 1	Cluster 2	Cluster 3			*p*	
	(*n* = 46)	(*n* = 65)	(*n* = 38)	(*n* = 340)			
	Mean ± SD	Mean ± SD	Mean ± SD	Mean ± SD			
	(95% CI ^†^)	(95% CI ^†^)	(95% CI ^†^)	(95% CI ^†^)			
**Maternal Health**							
Health Status ^2^	3.33 ± 0.85	3.22 ± 0.93	3.42 ± 0.98	3.46 ± 0.96	0.839	0.473	0.008
	(3.08, 3.58)	(2.99, 3.45)	(3.10, 3.74)	(3.36, 3.56)			
**Maternal Behaviors**							
Physical Activity Level ^3^	11.91 ± 8.67	13.14 ± 8.45	11.63 ± 7.63	14.74 ± 9.84	2.38	0.069	0.015
	(9.34, 14.49)	(11.04, 15.23)	(9.12, 14.14)	(13.69, 15.79)			
Screentime (minutes/day)	362.94 ± 229.95	422.08 ± 337.77	451.58 ± 396.60	329.47 ± 260.98	3.433	0.017	0.022
	(294.65, 431.22)	(338.38, 505.77)	(321.22, 581.94)	(301.63, 357.31)			
Fruit and Vegetable Intake (servings/day) ^4^	4.36 ± 1.64	4.41 ± 1.75	4.04 ± 2.02	4.54 ± 1.84	0.93	0.426	0.006
	(3.87, 4.85)	(3.97, 4.84)	(3.38, 4.71)	(4.35, 4.74)			
Sugar-Sweetened Beverage Intake (servings/day) ^4^	0.73 ± 0.61	0.96 ± 0.81	0.77 ± 0.88	0.69 ± 0.86	1.504	0.213	0.012
	(0.55, 0.91)	(0.76, 1.16)	(0.48, 1.06)	(0.60, 0.78)			
**Parenting Practices**							
Models Physical Activity through Co-Play with Child (days/week) ^5^	2.82 ± 1.61	3.31 ± 1.94	3.30 ± 1.75	3.83 ± 1.83	5.479	0.001 ^D^	0.033
	(2.34, 3.29)	(2.83, 3.79)	(2.73, 3.88)	(3.63, 4.02)			
Models Healthy Eating ^6^	3.57 ± 0.71	3.46 ± 0.80	3.35 ± 0.79	3.69 ± 0.77	3.255	0.022	0.022
	(3.36, 3.78)	(3.26, 3.66)	(3.09, 3.61)	(3.61, 3.77)			
Use Food to Reward Child’s Healthy Eating ^6^	2.27 ± 0.68	2.30 ± 0.73	2.68 ± 0.91	2.34 ± 0.73	2.771	0.041	0.017
	(2.07, 2.47)	(2.12, 2.48)	(2.39, 2.98)	(2.26, 2.42)			
Pressures Child to Eat ^6^	2.50 ± 0.84	2.38 ± 0.86	2.74 ± 1.01	2.20 ± 0.95	4.849	0.002 ^F^	0.030
	(2.25, 2.75)	(2.17, 2.60)	(2.40, 3.07)	(2.10, 2.30)			
Controls Child Food Intake Amounts ^6^	3.30 ± 0.61	3.07 ± 0.69	3.28± 0.86	3.05 ± 0.80	1.284	0.093	0.014
	(3.12, 3.49)	(2.89, 3.24)	(3.00, 3.56)	(2.96, 3.13)			
**Child Health Status ^2^**	4.07 ± 0.90	4.17 ± 0.86	4.21 ± 0.84	4.51 ± 0.72	6.189	<0.0001 ^DE^	0.046
	(3.80, 4.33)	(3.96, 4.38)	(3.93, 4.49)	(4.43, 4.58)			
**Child Behaviors**							
Child Physical Activity Level ^3^	23.28 ± 11.78	25.95 ± 11.18	25.66 ± 9.78	26.50 ± 11.58	1.128	0.337	0.007
	(19.78, 26.78)	(23.18, 28.72)	(22.44, 28.87)	(25.26, 27.73)			
Child Screentime (minutes/day)	300.00 ± 176.04	371.08 ± 324.96	325.66 ± 287.13	283.24 ± 269.85	1.857	0.136	0.012
	(247.72, 352.23)	(290.56, 451.60)	(231.28, 450.04)	(254.45, 312.02)			
Sugar-Sweetened Beverages ^4^	0.24 ± 0.34	0.47 ± 0.49	0.44 ± 0.45	0.28 ± 0.45	4.664	0.003 ^AE^	0.028
	(0.14, 0.34)	(0.35, 0.60)	(0.29, 0.58)	(0.24, 0.33)			
Milk ^4^	0.64 ± 0.46	0.87 ± 0.29	0.74 ± 0.37	0.84 ± 0.36	5.648	0.001 ^AD^	0.033
	(0.50, 0.77)	(0.80, 0.88)	(0.62, 0.86)	(0.80, 0.88)			
100% Fruit Juice ^4^	0.83 ± 0.34	0.64 ± 0.34	0.52 ± 0.38	0.53 ± 0.39	8.176	<0.0001 ^BD^	0.053
	(0.72, 0.93)	(0.56, 0.72)	(0.40, 0.65)	(0.49, 0.57)			

* df = Degrees of Freedom; # Analysis of Covariance (ANCOVA) with family affluence score as the covariate. Superscript capital letters indicate significant (*p* < 0.05) between-group differences using Bonferroni post-hoc tests: ^A^ Cluster 1 and Cluster 2, ^B^ Cluster 1 and Cluster 3, ^C^ Cluster 2 and Cluster 3, ^D^ Cluster 1 and White, ^E^ Cluster 2 and White, and ^F^ Cluster 3 and White. ^†^ CI = confidence interval. ^1^ Ward’s Hierarchical Cluster Analysis was used to assign Hispanic mothers to clusters based on acculturation (i.e., three personal measures [language of survey, language used in home, country of birth] and three environmental measures based on census tract [% foreign-born individuals, % foreign-born individuals arriving within the period 2010–2015, % Spanish-speaking households speaking English less than very well]). ^2^ The 5-point agreement rating: poor, fair, good, very good, excellent; scored 1 to 5, respectively; higher score indicates better health [2,3]. ^3^ Days/week engaged in walking, moderate activity, and vigorous activity weighted by exercise intensity (weights of 1, 2, 3, respectively) and summed to create a scale score; higher scale score indicates greater activity level. Possible score range = 0 to 42 [4,5,6]. ^4^ Higher score indicates greater servings consumed daily [6,7,8,9,10,11]. ^5^ Days/week mother engages in physical activity with child. Possible score range = 0 to 7. ^6^ The 5-point agreement rating: strongly disagree, disagree, neither agree nor disagree, agree, strongly agree; scored 1 to 5, respectively; scale score equals average of item scores; higher scale scores indicate greater expression of the trait. Possible score range = 1 to 5. Cronbach alphas for the 4-item Models Healthy Eating, 3-item Uses Food to Reward Child’s Healthy Eating, 4-item Pressures Child to Eat, and 4-item Controls Child Food Intake Amounts scales are 0.71, 0.75, 0.66, and 0.66, respectively.

**Table 5:** Home Environment Characteristics (*n* = 489).

Characteristic	Hispanic Mothers’ Clusters ^1^			White Mothers	*F df* = *3, 485* *	ANCOVA #	Partial Eta-Squared
	Cluster 1	Cluster 2	Cluster 3	(*n* = 340)		*p*	
	(*n* = 46)	(*n* = 65)	(*n* = 38)				
	Mean ± SD	Mean ± SD	Mean ± SD	Mean ± SD			
	(95% CI ^†^)	(95% CI ^†^)	(95% CI ^†^)	(95% CI ^†^)			
**Family Mealtime**							
Importance Placed on Family Meals ^2^	4.54 ± 0.54	4.37 ± 0.73	4.41 ± 0.73	4.48 ± 0.63	0.765	0.514	0.005
	(4.38, 4.70)	(4.19, 4.56)	(4.17, 4.65)	(4.41, 4.54)			
Family Meals (meals/week)	10.24 ± 5.65	12.37 ± 4.44	12.92 ± 4.75	12.88 ± 4.59	3.669	0.012	0.026
	(8.56, 11.92)	(11.27, 13.47)	(11.36, 14.48)	(12.39, 13.37)			
Family Meal Location (days/week)							
Less Healthy Locations	7.70 ± 5.52	4.35 ± 3.09	4.03 ± 3.76	2.97 ± 3.41	18.825	0.000 ^ABD^	0.128
	(6.06, 9.34)	(3.59, 5.12)	(2.79, 5.26)	(2.60, 3.33)			
Car	2.76 ± 3.05	0.46 ± 1.03	0.39 ± 0.86	0.36 ± 1.02	42.79	<0.0001 ^ABD^	0.215
	(1.86, 3.67)	(0.21, 0.72)	(0.11, 0.68)	(0.25, 0.47)			
Fast Food Restaurant	1.39 ± 1.73	1.20 ± 1.23	1.39 ± 1.44	0.70 ± 1.08	9.123	<0.0001 ^DEF^	0.053
	(0.88, 1.91)	(0.90, 1.50)	(0.92, 1.87)	(0.59, 0.82)			
Front of TV	3.54 ± 2.77	2.69 ± 2.41	2.24 ± 2.17	1.90 ± 2.38	5.594	0.001 ^D^	0.044
	(2.72, 4.37)	(2.10, 3.29)	(1.52, 2.95)	(1.65, 2.16)			
Healthy Location: Dining Table	2.54 ± 2.80	4.18 ± 2.43	5.66 ± 1.70	5.08 ± 2.29	15.315	<0.0001 ^ABCD^	0.107
	(1.71, 3.38)	(3.58, 4.79)	(5.10, 6.22)	(4.83, 5.32)			
**Household Food Availability (servings/person/day) ^3^**							
Fruit/Vegetables	5.79 ± 2.29	5.93 ± 2.16	5.35 ± 2.19	6.03 ± 2.03	1.552	0.216	0.008
	(5.11, 6.46)	(5.40, 6.47)	(4.63, 6.07)	(5.81, 6.25)			
Sugar-Sweetened Beverages	0.22 ± 0.19	0.28 ± 0.24	0.28 ± 0.26	0.22 ± 0.26	1.258	0.241	0.009
	(0.17, 0.28)	(0.22, 0.34)	(0.19, 0.36)	(0.19, 0.25)			
**Home Physical Activity Environment**							
Indoor/Home Space and Supports ^2^	2.93 ± 1.04	3.18 ± 0.80	3.21 ± 1.00	3.39 ± 0.80	4.235	0.008 ^D^	0.030
	(2.62, 3.24)	(2.98, 3.38)	(2.88, 3.54)	(3.31, 3.48)			
Outdoor/Yard Space and Supports ^2^	3.91 ± 0.91	4.32 ± 0.62	4.33 ± 0.89	4.45 ± 0.59	6.144	<0.0001 ^AD^	0.048
	(3.60, 4.23)	(4.14, 4.49)	(4.02, 4.63)	(4.39, 4.51)			
Neighborhood Space and Supports ^2^	3.66 ± 1.08	3.76 ± 0.86	3.90 ± 1.19	4.12 ± 0.99	1.604	0.029	0.029
	(3.34, 3.98)	(3.55, 3.98)	(3.51, 4.10)	(4.02, 4.23)			
Neighborhood Safety ^2^	3.07 ± 0.90	3.02 ± 0.79	3.57 ± 0.81	3.50 ± 0.85	4.548	<0.0001 ^BDE^	0.053
	(2.80, 3.33)	(2.82, 3.21)	(3.30, 3.83)	(3.41, 3.60)			

* df = Degrees of Freedom; # Analysis of Covariance (ANCOVA) with family affluence score as the covariate. Superscript capital letters indicate significant (*p* < 0.05) between-group differences using Bonferroni post-hoc tests: ^A^ Cluster 1 and Cluster 2, ^B^ Cluster 1 and Cluster 3, ^C^ Cluster 2 and Cluster 3, ^D^ Cluster 1 and White, ^E^ Cluster 2 and White, and ^F^ Cluster 3 and White. ^†^ CI = confidence interval; ^1^ Ward’s Hierarchical Cluster Analysis was used to assign Hispanic mothers to clusters based on acculturation (i.e., three personal measures [language of survey, language used in home, country of birth] and three 3 environmental measures based on census tract [% foreign-born individuals, % foreign-born individuals arriving within the period 2010–2015, % Spanish-speaking households speaking English less than very well]). ^2^ The 5-point agreement rating: strongly disagree, disagree, neither agree nor disagree, agree, strongly agree; scored 1 to 5, respectively; scale score equals average of item scores; higher scale scores indicate greater expression of the characteristic. Possible score range = 1 to 5. Cronbach alphas for the scales were as follows: Indoor/Home Space and Supports for Physical Activity = 0.71; Outdoor/Yard Space and Supports for Physical Activity = 0.74; Neighborhood Space and Supports for Physical Activity = 0.42, and Neighborhood Safety = 0.42. ^3^ Higher score indicates greater servings available daily per household member [6,7,8,9,10,11].

## Data Availability

Data sharing not applicable. No new data were created or analyzed in this study. Data sharing is not applicable to this article.
